# Bioengineering Interventions to Enhance the Capacity of the Gut Microbiota in Controlling Food Allergies

**DOI:** 10.3390/life16030433

**Published:** 2026-03-07

**Authors:** Manish Kumar, Shivani Nalla, Jatindra N. Tripathy, Akhilesh Kumar Shakya

**Affiliations:** 1 Department of Civil, Environmental and Construction Engineering, Texas Tech University, Lubbock, TX 79409, USA; maniskum@ttu.edu; 2 Center for Biotechnology & Genomics, Texas Tech University, Lubbock, TX 79409, USAjatindra.n.tripathy@ttu.edu (J.N.T.); 3 Department of Chemical Engineering, Texas Tech University, Lubbock, TX 79409, USA; 4 Bioengineering, Whitacre College of Engineering, Texas Tech University, Lubbock, TX 79409, USA

**Keywords:** food allergies, peanut allergies, milk allergies, egg allergies, probiotics, recombinant probiotics, allergen-specific immunotherapy, oral immunotherapy, gut microbiota, polyphenols, carbohydrates

## Abstract

Food allergies arise when environmental factors, lifestyle choices, and genetic predispositions affect the integrity of the gut epithelial barrier. Under healthy conditions, gut microbiota supports intestinal tight junction integrity and promotes immune tolerance to dietary allergens. Disruption of this microbiota increases susceptibility to epithelial barrier leakage, thereby enabling food allergens to penetrate the bloodstream from the gut and leading to allergic sensitization. Restoring gut homeostasis through allergen-specific immunotherapy (AIT), executed via oral termed as oral immunotherapy (OIT), skin as subcutaneous immunotherapy (SCIT), or under the tongue in the form of sublingual immunotherapy (SLIT), remains a promising yet complex and multifaceted approach. In parallel, probiotics offer a simpler alternative to reinforce epithelial barrier function, restore cellular homeostasis, mitigate allergy symptoms, and represent the probiotics-based OIT. Recently, several bioengineering strategies have been developed toward enriching gut microbiota, such as using additives such as carbohydrates, polyphenols, and probiotics. While generic probiotics have shown efficacy, their undefined dosages and administration protocols pose challenges for clinical standardization in the form of OIT. Emerging developments include recombinant probiotics engineered to express the specific allergen in a controlled manner inside the gut. However, safety concerns regarding their clinical application remain under active discussion. This review highlights various bioengineering strategies to enhance the probiotic capacity, address safety considerations, and explore future prospects for managing food allergies.

## 1. Introduction

**Figure 1:**
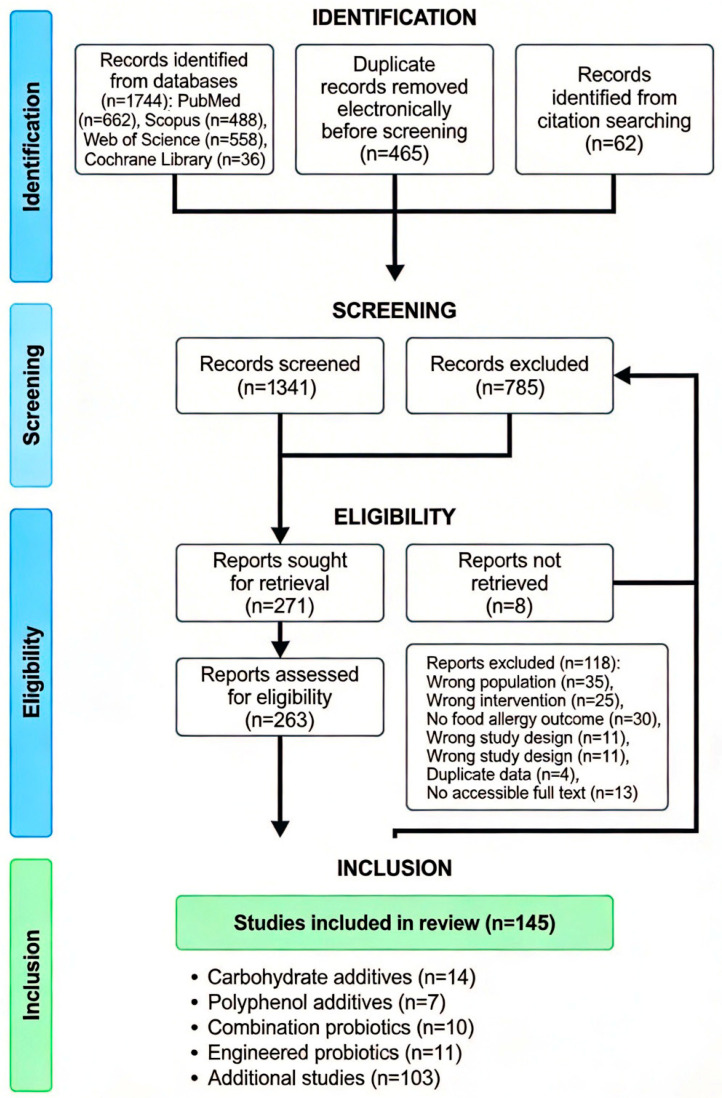
PRISMA flow diagram for systematic review. The literature search yielded 1744 records from databases (PubMed, Scopus, Web of Science, and Cochrane Library) and 62 from a citation search. After removing 465 duplicates, 1341 records were screened. Of 263 full-texts assessed, 145 studies met criteria: 14 on carbohydrate additives, 7 on polyphenol additives, 10 on combination probiotics, 11 on engineered probiotics, and 103 on other interventions [5].

Food allergies have become a significant global health concern. Before the mid-20th century, they were not widely recognized as a distinct medical condition. Over time, increased awareness and advances in research have greatly improved the ability to diagnose, treat, and manage food allergies [1]. The global prevalence is estimated at 4.3%, although rates vary by region. In developed countries, food allergies affect roughly 8% of children and 10% of adults population. Notably, North America and Northern Europe report higher incidences of peanut and tree nut allergies, which are among the leading causes of long-term food-induced allergic anaphylaxis [2,3,4]. This comprehensive review systematically evaluated bioengineered interventions targeting gut microbiota to control food allergies. Following PRISMA guidelines, we analyzed 145 studies encompassing carbohydrate additives, polyphenol compounds, combination probiotics, and engineered probiotic strains (Figure 1) [5].

Food allergy management, as outlined by the American Academy of Allergy, Asthma & Immunology and the American College of Allergy, Asthma, & Immunology (AAAAI/ACAAI) joint task force, with an allergy-focused history, targeted testing like skin prick testing and detection of systemic allergen-specific immunoglobulins E (IgE), and, to finally confirmation include a physician-supervised oral food challenge, which is considered the current gold standard for food allergies diagnosis [1,6,7]. Care plans to manage food allergies widely focus on patient education, supervision of dietary management, clear, specific food avoidance strategies, and emergency preparedness, such as the use of auto-injectors (typically two) at the time of a life-threatening event like anaphylaxis, and describe observation and follow-up after reactions [8,9,10,11,12]. Temporary control of allergy symptoms can be achieved with antihistamines and anti-IgE drugs; however, only allergen-specific immunotherapy (AIT), a disease-modifying treatment that repeatedly exposes the immune system to small amounts of the allergen over an extended period, gradually inducing tolerance to that specific allergen, is considered a potential long-term approach to managing food allergies [13,14,15].

Traditional subcutaneous allergen injection at specific intervals in the form of subcutaneous immunotherapy (SCIT), requiring 50–80 allergen injections in varying allergen doses over 3–5 years, is approved for desensitization of airway allergies, but it is not approved for food allergies due to severe side effects [15,16,17]. Recently, alternative skin-based approaches, such as microneedles and PULSE patches, have demonstrated effective therapeutic efficacy in preclinical studies [17,18,19,20]. However, adverse reactions, manufacturing scale-up barriers, variability in performance, and reliance on specialized instrumentation continue to hinder advancement to clinical stages [18]. The OIT, which involves the controlled ingestion of an allergen at a specific dose and frequency, can induce partial desensitization; however, allergen avoidance and access to epinephrine remain necessary with current OIT protocols [18,21,22]. For instance, OIT functions effectively for peanut, milk, and egg allergies [23,24,25], yet it is associated with risks such as dose-related adverse reactions and eosinophilic esophagitis. Therefore, adjunct anti-IgE therapy may improve safety and the rate of desensitization [26]. Recently, the FDA approved Omalizumab, an antibody-based treatment that mitigates food allergy symptoms, although it also carries potential side effects [26,27,28].

Food allergies arise at the intersection of barrier biology, immune learning, and daily exposures to allergens [29]. The pathogenesis of food allergies extends beyond genetic predisposition and immediate immunological reactions to encompass profound epigenetic alterations that shape immune cell differentiation, activation, and tolerance acquisition [30]. Epigenetic mechanisms, primarily DNA methylation and histone modifications, mediate gene–environment interactions, translating environmental exposures, dietary factors, and microbial signals into heritable changes in gene expression patterns without changing the underlying DNA sequence [31]. DNA methylation, the covalent addition of methyl groups to cytosine residues at CpG dinucleotides, has been studied extensively for epigenetic modification in food allergy pathogenesis [31]. This modification typically occurs in gene promoters, where increased methylation restricts access by transcription factors, leading to transcriptional silencing. Conversely, DNA demethylation increases chromatin accessibility and promotes gene expression [32]. These reversible modifications play pivotal roles in establishing and maintaining the Th2-skewed immune profile characteristic of food allergies [33,34].

Sensitization against the specific food can begin even before the first full serving of a food reaches the gut: damaged skin in eczema can permit entry of food proteins, while airborne or dust-borne particles can further prime the immune system through the airways [35,36,37]. After this priming phase, ingestion of the same allergen activates IgE-bound mast cells within a T helper 2 (Th2)-skewed immune environment, triggering symptoms that can include allergic anaphylaxis [38,39]. This response is shaped not only by the allergen itself but also by the context in which the host initially ‘learned’ about it, including epithelial barrier integrity and the nature of mucosal immune conditioning [40]. Building on this, the gut acts as both a gatekeeper and a guide. A well-organized intestinal microbiota helps maintain intestinal tight junctions, provides metabolites that reduce unnecessary inflammation, and promotes regulatory T cells (Tregs) that support tolerance rather than reactivity [41,42,43]. Conversely, losing key fermenters or reducing diversity weakens the barrier function and limits the immune repertoire, thereby increasing the risk that harmless dietary proteins will be mistaken for threats [43]. This conceptual framework, which integrates barrier and instructional strategies, makes microbiota enrichment a viable therapeutic approach for food allergies.

## 2. Allergic Immune Modulation Through Gut Microbiota

The intestinal barrier plays a vital role in facilitating nutrient and fluid absorption and preventing the entry of harmful substances, including toxins and pathogens [44]. Key components of this barrier include the gut microbiota and the mucus layer, both of which are essential for modulating immune responses and protecting against pathogen invasion. Thus, preserving the integrity and function of the mucosal barrier is vital for overall health and survival [45]. In individuals with allergies, this barrier is often compromised, thereby increasing intestinal permeability to allow food allergens to leak from the intestinal lumen to the bloodstream [46]. Moreover, disruptions in the mucus layer can lead to the formation of micro-openings, which allow allergens to traverse the epithelial barrier [46]. The gut microbiota in allergic individuals elicits a distinct immune response compared to that of non-allergic individuals [47]. In allergic cases, the immune system erroneously identifies harmless substances—such as pollen, peanuts, eggs, shellfish, wheat, and soy—as threats [48]. At the time of dysbiosis, an imbalance in gut microbiota establishes a pro-inflammatory environment that undermines tolerance by activating the NOD-, LRR- and pyrin domain-containing protein 3 (NLRP3) inflammasome, a cytosolic innate immune sensor that integrates microbial, metabolic, and sterile danger signals [49]. Under homeostatic conditions, physiological NLRP3 activation protects against pathogens, promotes intestinal epithelial repair, and supports Tregs differentiation, thereby maintaining immune tolerance to commensal flora and dietary antigens/allergens [50]. However, dysbiosis-induced alterations in microbial metabolite profiles trigger aberrant NLRP3 inflammasome assembly and sustained caspase-1 activation, leading to excessive production of the pro-inflammatory cytokines IL-1β and IL-18, which directly amplify Th2-driven allergic responses [51,52]. In experimental models of allergic inflammation, NLRP3 activation in alveolar macrophages and dendritic cells (DCs) promotes inflammatory cell recruitment, mucus hypersecretion, and polarization of a type 2 immune response, and genetic ablation or pharmacological inhibition of NLRP3 significantly attenuates allergic sensitization and anaphylaxis [49]. The dysbiosis–NLRP3 axis operates through multiple molecular mechanisms: *(a)* decreased production of short-chain fatty acids (SCFAs) removes physiological brakes on inflammasome activation, *(b)* increased intestinal permeability permits translocation of lipopolysaccharide and bacterial DNA that prime and activate NLRP3 via toll like receptors (TLR)4 and TLR9, and *(c)* loss of protective commensal bacteria eliminates metabolites that normally suppress reactive oxygen species production and *(d)* mitochondrial dysfunction, both canonical triggers of NLRP3 assembly [53,54,55]. Paradoxically, complete NLRP3 deficiency also promotes dysbiosis, as knockout mice exhibit mucus layer thinning, increased intestinal permeability, overproliferation of pathogenic Enterobacteriaceae, and decreased Lactobacillus abundance [56]. The short chain fatty acids (SCFAs) exert context-dependent effects on NLRP3: SCFA butyrate specifically inhibits endothelial and epithelial NLRP3 inflammasome activation through antioxidant mechanisms and suppression of reactive oxygen species production under steady-state conditions, but can paradoxically activate NLRP3 in macrophages under inflammatory conditions through histone deacetylase inhibition that prevents transcription of the anti-apoptotic gene CFLAR and the anti-inflammatory cytokine interleukin (IL)-10 [57,58]. These findings establish NLRP3 as a critical molecular rheostat that, when dysregulated by dysbiosis, shifts the mucosal immune system from tolerance toward pro-allergic inflammation, providing a direct mechanistic link between altered microbial metabolites and the inflammatory milieu that predisposes to food allergy development [59,60,61].

Genome-wide DNA methylation profiling has provided extensive epigenetic dysregulation of genes governing T-cell receptor signaling, metabolic regulation, and inflammatory responses in individuals with food allergy. Naive CD4+ T cells from infants with food allergy exhibit hypo-responsiveness to activation, attributable to differential methylation of genes encoding critical signaling molecules, including regulatory-associated protein of mTOR (RPTOR), phosphatidylinositol 3-kinase delta (PIK3D), mitogen-activated protein kinase 1 (MAPK1), and forkhead box O1 (FOXO1) [31,62]. Methylation alterations at 92 CpG loci spanning 49 genes were significantly associated with food allergy, and gene ontology enrichment analyses implicated mitogen-activated protein kinase (MAPK) signaling pathways. Suboptimal CD4+ T-cell development and activation, mediated by dysregulated DNA methylation of MAPK-associated genes, predispose to aberrant immune responses to food allergens during early childhood [31,62].

**Figure 2:**
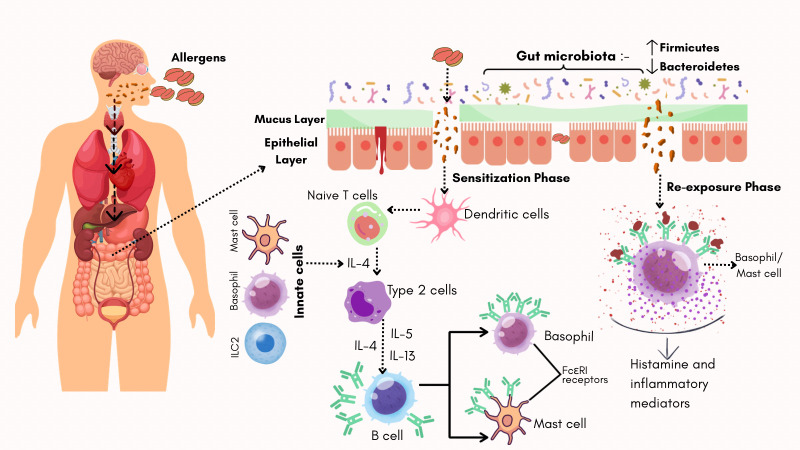
Illustration of the mechanism of food allergy in an allergy-prone individual, depicting the sensitization and re-exposure phases. During the sensitization phase, food allergens activate dendritic cells, triggering a Type 2-driven response that produces allergen-specific IgE antibodies. These IgE antibodies bind to FcεRI receptors on the surface of mast cells and basophils, conditioning the immune system without causing symptoms. Upon re-exposure, the food allergen binds IgE on mast cells and basophils, triggering the release of histamine and other inflammatory mediators that cause allergic symptoms [69,70].

In allergic subjects, once allergens break the epithelial barrier and enter the bloodstream, they are captured by antigen-presenting cells (APCs), such as dendritic cells (DCs). These cells migrate to the lymph nodes and present allergens to naïve T cells [63]. In response, T cells secrete cytokines such as interleukin-4 (IL-4), which drive their differentiation into Th2 cells. IL-4 is also produced by innate immune cells, including basophils, mast cells, and type 2 innate lymphoid cells [64]. This cell signaling cascade amplifies the release of other key cytokines, including IL-4, IL-5, and IL-13, which mediate allergic inflammation [65,66,67]. This stage is known as allergen sensitization, in which the immune system is primed to respond to specific allergens. Upon subsequent exposure to the allergen, allergen-specific IgE antibodies are produced by plasma B cells whichbind to receptors on effector cells such as mast cells and basophils, leading to their activation.. This activation triggers the release of histamine and other inflammatory mediators, leading to allergic symptoms [68]. This phase is referred to as the allergen challenge (Figure 2) [69,70]. Therefore, enhancing the gut microbiota is a promising strategy for inducing immune tolerance and mitigating food allergy symptoms.

Strengthening the intestinal barrier requires coordinated microbiota-mediated effects on tight junction proteins, mucus layer homeostasis, and epithelial integrity, mediated by microbial metabolites (butyrate, propionate, and bile acids). These metabolites shared immunoregulatory mechanisms: reinforcement of epithelial tight junctions and mucus layer integrity to prevent allergen translocation, education of DCs toward tolerogenic phenotypes that favor regulatory over effector T-cell differentiation, and metabolic reprogramming of T cells to suppress pro-allergic Th2 responses while enhancing IL-10-producing Treg populations [32,71]. The spatial distribution and temporal dynamics of these metabolites create distinct immunological profiles along the intestinal tract, with the highest SCFAs concentrations in the colon, bile acid signaling dominating in the ileum and proximal colon, and tryptophan metabolite effects concentrated at barrier surfaces where microbial communities interface with host tissues [72,73]. Collectively, these microbiota-derived metabolites function as essential mediators that translate microbial composition into immunological outcomes, determining whether the mucosal immune system interprets food antigens as harmless nutrients that require tolerance or threats that elicit inflammatory responses.

## 3. Strategies to Enhance the Gut Microbiota for Management of Food Allergies

**Figure 3:**
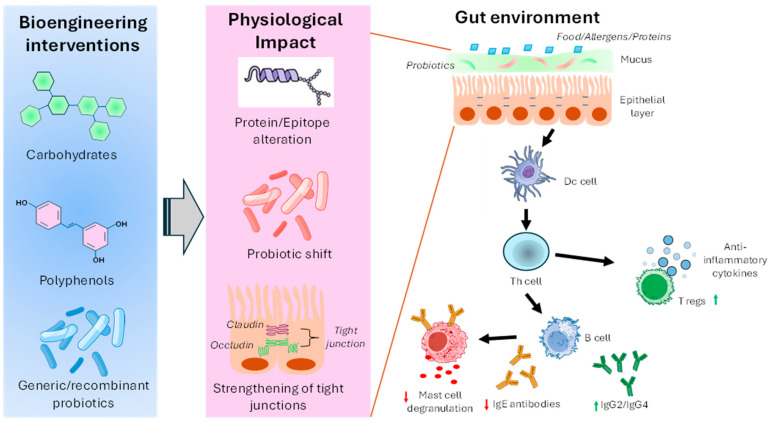
Schematic illustration of various bioengineering interventions and their physiological effects on the gut environment to modulate allergic immune responses against food allergens.

Over the decades, significant progress has been made in developing several bioengineering approaches to enrich probiotic populations and improve gut health to combat food-induced hypersensitivity (Figure 3).

### 3.1. Prebiotics

Prebiotics, defined as nondigestible food components that specifically promote the growth or activity of beneficial gut microbes, work through substrate-specific fermentation pathways that alter both the microbial community structure and its metabolic functions [74]. Diets rich in diverse plant fibers like carbohydrates, polyphenols nourish beneficial microbes, while prebiotics and carefully selected probiotics provide additional support; synbiotics (a combination of both) strengthen the microbial ecosystem [75,76].

#### 3.1.1. Carbohydrate Additives

The biological activity of carbohydrates is profoundly impacted by their chemical structure. Structural modifications and chemical alterations, such as sulfation, acetylation, and phosphorylation, enhance their physicochemical properties and bioactive functions. These chemical modifications enable carbohydrates to interact more effectively with immune cells and the gut microbiota, thereby improving their efficacy in combating food allergies [77,78,79]. Table 1 lists several polysaccharides and oligosaccharides, classified as prebiotics, that have been investigated as supplements to enhance gut immunity and manage food allergies. Among several, chitin- and chitosan-based polysaccharides exemplify this well. Across multiple food-allergy models, chitin- and chitosan-based derivatives enhance the capacity to mitigate allergic clinical reactions, suppress allergen-specific IgE and Th2 cytokines, and shift the immune response toward a Th1/regulatory profile [80,81,82]. They even function as delivery carriers, such as in DNA-loaded chitosan nanoparticles, to transport tolerogenic signals like TGF-β to the gut, and are also effective additives for enriching gut microbiota [83]. Marine- and plant-derived fibers show similar effects. For instance, red-edge tea polysaccharides (RETPSs) [84], ulva polysaccharides [85], depolymerized sulfated galactans from Eucheuma [86], fucoidan [87], inulin [88], and laminarin [89] from different sources have all been shown to alleviate allergic symptoms and suppress mast-cell mediators, and promote a microbiota shift [88]. Inulin-type fructans are fermented by Bifidobacterium and Lactobacillus species, which promote the production of lactate and SCFAs, including acetate, propionate, and butyrate, with the relative proportions determined by substrate chain length, microbial composition, and host age [90].

Besides the polysaccharides, small oligosaccharides provide adequate enrichment for probiotics via providing carbon sources for saccharolytic bacteria, particularly the Bifidobacterium and Lactobacillus species, while simultaneously restricting the growth of pathogenic taxa [91]. Prebiotic administration increases alpha diversity and shifts beta diversity toward compositional profiles characteristic of healthy, disease-free states, with inulin and fructo-oligosaccharides (FOSs) showing the strongest bifidogenic effects [92]. Mechanistically, these oligosaccharides are transported into bacterial cells via specialized ATP-dependent cassette transporters and glycoside hydrolases encoded in polysaccharide utilization loci, where they undergo sequential enzymatic degradation into monosaccharides that enter glycolytic and fermentative pathways [93]. The resulting metabolic flux generates SCFAs, including acetate, propionate, and butyrate, that affect microbial community composition and substrate type [94]. Moreover, prebiotics induce functional changes in the gut metagenome, enriching genes associated with carbohydrate metabolism, particularly phosphate acetyltransferase, malate dehydrogenase, propionate kinase, acetate kinase, and acetaldehyde dehydrogenase, which collectively enhance the metabolic capacity for SCFA synthesis [95].

Different types of oligosaccharides that serve as prebiotics displayed differential fermentation kinetics [96]. For instance, FOSs predominantly produce acetate and lactate SCFAs during early fermentation (0–12 h), followed by conversion to propionate and butyrate SCFAs (12–48 h), whereas galacto-oligosaccharides (GOSs) generate more sustained butyrate production through selective enrichment of butyrate-producing Firmicutes [94]. Similarly, GOSs display strain- and degree-of-polymerization-specific consumption patterns by bifidobacteria: *Bifidobacterium longum* subsp. infantis preferentially consumes GOS tetrasaccharides and pentasaccharides (DP 4-5), *Bifidobacterium breve* targets higher-molecular-weight oligosaccharides (DP 6-8), while *Bifidobacterium adolescentis* exhibits broader substrate utilization spanning (DP 3-8) [97]. Purified GOS fractions demonstrate selectivity indices of 2.1–3.0 for bifidobacteria over total anaerobes under in vitro fermentation conditions, confirming their bifidogenic specificity [98]. Overall, carbohydrate additives function not merely as generic prebiotics but as specific fermentable substrates that microbial communities convert into defined metabolite profiles with distinct immunomodulatory properties.

**Table 1:** A list of polysaccharides and oligosaccharides and their role in enhancing gut microbiota to mitigate the symptoms of food allergies.

Polysaccharides/Oligosaccharides	Food Allergy	Outcome	Reference
Chitosan	Shrimp tropomyosin, Ovalbumin, Peanut	Chitosan oligosaccharide (COS) alleviated shrimp tropomyosin allergy symptoms; reduced IgE, histamine, and Th2 cytokines; and enhanced the Th1 response.	[81,82,83]
		Chitosan nanoparticles (carrying TGF-β DNA) increased intestinal TGF-β and reduced ovalbumin-induced allergy symptoms after oral delivery.	
		Chitin/Chitosan protected against peanut-induced reactions by reducing IgE levels and Th2 cytokines.	
Red-edge tea polysaccharide (RETPS)	Ovalbumin	RETPS-3 and RETPS-4 from red-edge tea reduced Ovalbumin-specific IgE, histamine, and mast cell protease levels, alleviated allergic symptoms (itching, hypothermia, and diarrhea), and improved the gut microbiota.	[84]
Ulva-derived polysaccharides	Ovalbumin	Ulva-derived compounds alleviated allergic symptoms by lowering IgG1 and Ovalbumin-specific IgE, increasing protective IgG, promoting Th1 immunity, improving microbial balance, and strengthening the intestinal barrier.	[85]
Depolymerized sulfated galactans	Ovalbumin	Oral depolymerized sulfated galactans suppressed anaphylaxis and inflammatory markers, while gut tissues showed reduced epithelial damage, fewer inflammatory cells, and improved structure.	[86]
Fucoidan sulfated polysaccharide	Ovalbumin	Fucoidan suppressed allergic responses by reducing Ovalbumin-specific IgE, histamine, mast cell protease-1, and Th2 cytokines, while increasing IL-10 and TGF-β, and improving the gut microbiota.	[87]
Inulin	Ovalbumin	Inulin-based treatment reduced anaphylaxis and diarrhea, stabilized temperature, lowered Th2 markers (IL-4, IL-5, IL-13, IgE, and IgG1), enhanced Th1/regulatory responses, and restored ileal microbiota by increasing beneficial bacteria	[88]
*Porphyra haitanensis* sulfated polysaccharides	Ovalbumin	Marine sulfated polysaccharides (PHP40, PHP80) restored microbial balance, boosted beneficial bacteria (Bacillus, Enterococcus), suppressed harmful ones (Staphylococcus), and enhanced galactose metabolism and bile acid biosynthesis.	[99]
Aloe	Ovalbumin	Processed aloe reduced allergic symptoms by suppressing Th2 cells, eosinophils, and mast cells, while boosting regulatory T cells and IL-10.	[100]
*Gracilaria lemaneiformis* polysaccharide	Ovalbumin	*Gracilaria lamaneiformis* fermented with *Lactobacillus acidophilus* lowered Ovalbumin-specific IgE, histamine, and mast cell protease, suppressed Th2/IL-4, and boosted Treg cells and IL-10.	[101]
Fructo-oligosaccharides (FOSs)	Peanut	FOSs modulated DCs in peanut-allergic individuals, promoting immune tolerance rather than allergic inflammation.	[102]
Sulphated oligosaccharides	Ovalbumin	Sulfated oligosaccharides from *Gracilaria lamaneiformis* promote Tregs, suppressing Th2-driven IgE production and mast cell mediator release.	[103]
Combined intake of short fructan (1-kestose) and long fructan (inulin)	Ovalbumin	A combination of short- and long-chain fructans reduced allergy-related symptoms, such as diarrhea, and enhanced the gut microbiota.	[104]
Combination of short- and long-chain fructo-oligosaccharides (scFOSs/lcFOSs)	Peanut	scFOSs/lcFOSs reduced mast cell activity, boosted Tregs, and enhanced beneficial gut bacteria through prebiotic supplementation.	[105]
Lactulose	Cow milk	Synbiotic administration increased gut butyrate and markedly reduced allergic responses, including anaphylaxis, in mice.	[106]

#### 3.1.2. Polyphenols Additives

Polyphenols provide a complementary strategy to boost the health of gut microbiota by modifying allergen surfaces and reducing oxidative stress at mucosal sites [107] (Table 2). Compounds such as bisdemethoxycurcumin suppress Th2 cytokines, decrease mast cell activity, and enhance regulatory and Th1 responses [108]. Interestingly, covalent binding of Epigallocatechin gallate (EGCG) or chlorogenic acid to shrimp tropomyosin or the peanut allergen Ara h1 reduces IgE recognition and mast cell degranulation, producing fewer reactive fragments after digestion and limiting triggers for allergic reactions [109,110]. Similarly, apple condensed tannins and fruit–polyphenol complexes, when combined with peanut proteins, inhibit IgE binding and reduce allergen challenge responses [111]. Other polyphenols like anthocyanins undergo bacterial ring-fission and C-ring degradation to yield phloroglucinol derivatives from the A-ring and benzoic acids from the B-ring [112]. Polyphenols exert prebiotic-like effects through a dual mode of action termed “duplibiotic” activity, encompassing both antimicrobial and growth-promoting properties. These bioactive compounds, which reach the colon largely intact due to limited upper gastrointestinal absorption, undergo extensive biotransformation by specialized gut bacteria equipped with polyphenol-associated enzymes (PAZymes), including tannases, quercetinases, gallate decarboxylases, and phenolic acid decarboxylases. The enzymatic degradation of polyphenols generates low-molecular-weight phenolic metabolites such as 4-hydroxyphenylacetic acid (4-HPAA), protocatechuic acid, and various hydroxyphenylpropionic acids, which are more readily absorbed and exhibit distinct bioactivities [113,114]. Moreover, the antimicrobial activity of polyphenols selectively inhibits pathogenic species by disrupting bacterial cell membrane integrity, interfering with nucleic acid synthesis, altering cell wall composition, and inhibiting biofilm formation. This ecological disruption frees niches that are subsequently colonized by beneficial bacteria, particularly *Akkermansia muciniphila*, Lactobacillus spp., Bifidobacterium spp., *Faecalibacterium prausnitzii*, Lachnospiraceae, and Roseburia spp. Notably, another polyphenol, epigallocatechin gallate, has been shown to significantly increase Akkermansia abundance, which in turn promotes SCFAs like acetate and propionate production through nutritional cross-feeding with butyrate-producing bacteria. Similarly, fisetin supplementation enhances Lachnospiraceae abundance, a family strongly associated with elevated SCFA production and neuroprotective effects [114,115]. Polyphenol fermentation by these enriched bacterial populations substantially increases SCFA production [116]. *Lonicera caerulea* polyphenols have been shown to restore SCFA concentrations to physiological levels in dysbiotic states, and fermented polyphenol preparations are more effective than unfermented counterparts [117]. The mechanism involves selective enrichment of SCFA-producing taxa through both direct utilization of polyphenol catabolites as metabolic substrates and indirect microbiome restructuring that favors syntrophic microbial networks [114].

**Table 2:** A list of polyphenols and their role in enhancing gut microbiota to mitigate the symptoms of food allergies.

Polyphenols	Food Allergy	Outcome	Reference
Bisdemethoxycurcumin (BMDC)	Ovalbumin	BDMC treatment reduced allergic symptoms (anaphylaxis, diarrhea), improved intestinal health, suppressed Th2 responses, enhanced Th1 and regulatory responses, and inhibited inflammatory pathways, including MAPK and NF-κB.	[108]
Resveratrol	Ovalbumin	Resveratrol reduced IgE levels and cytokines, including IL-4 and IL-13.	[118]
Theaflavins	Ovalbumin	Theaflavins help prevent food allergy symptoms.	[119]
Two dietary polyphenols, Epigallocatechin gallate (EGCG) and Chlorogenic acid (CA)	Tropomyosin	Modifying shrimp tropomyosin with EGCG or chlorogenic acid changes its structure. These changes make it harder for the immune system by reducing the avidity with which IgE and IgG antibodies bind to it, thereby lowering its allergenic potential.	[107]
Apple condensed tannins (ACTs)	Ovalbumin	Mice treated with ACT had significantly lower Ovalbumin-specific IgE and IgG1 levels, thereby inhibiting the immune system’s response to food proteins.	[111]
EGCG and chlorogenic acid	Arah1 (peanut)	Modified Ara h1 with EGCG and cholorogenic acid reduced peanut allergenicity.	[109]
Polyphenol aggregation	Peanut	Mice administered peanut–polyphenol mixtures, particularly at higher polyphenol doses, had lower IgE levels.	[120]

#### 3.1.3. Combination of Carbohydrates and Polyphenols

The combined administration of polyphenols and oligosaccharides produces synergistic effects that exceed the sum of their individual actions [114]. This synergy operates through multiple complementary mechanisms: *(a)* polyphenols create favorable ecological conditions by selectively eliminating competing pathogenic bacteria and reducing oxidative stress, thereby allowing oligosaccharide-responsive beneficial taxa to flourish; *(b)* oligosaccharides provide readily fermentable carbon sources that sustain the growth of polyphenol-metabolizing bacteria; *(c)* both substrates, independently and cooperatively, enhance mucin production, creating additional niches for mucin-degrading beneficial bacteria such as *Akkermansia muciniphila* [121]. This substrate-level perspective reframes dietary interventions from generic prebiotic supplementation to precision fermentation substrate provision, in which specific molecular structures dictate microbial consumption patterns, metabolite profiles, and downstream immunological outcomes in food allergy prevention [122,123]. The microbial community shifts induced by this synbiotic combination lead to increased SCFAs production, enhanced microbial diversity, and strengthened gut barrier integrity [114]. Specifically, the interaction promotes expansion of Lactobacillus and Bifidobacterium populations capable of metabolizing both polyphenols and oligosaccharides, while simultaneously enriching *Faecalibacterium prausnitzii* and other butyrate-producing taxa that benefit from the metabolic intermediates generated by primary fermenters [121,124]. The anti-allergic efficacy of combined polyphenol and oligosaccharide supplementation arises from the integration of multiple hierarchical mechanisms operating at the microbial, metabolic, epithelial, and immunological levels. At the microbial level, polyphenols reshape the ecological landscape through selective antimicrobial activity and provision of metabolizable substrates, while oligosaccharides provide fermentable carbon that sustains beneficial populations. This restructured microbiome generates elevated SCFA concentrations, which serve as signaling molecules at the epithelial interface [125,126]. At the epithelial level, SCFAs like butyrate enhance barrier integrity by regulating tight junction proteins via the suppression of oxidative stress through activation of NOTCH signaling [127,128].

The multilayer interactions among polyphenols, oligosaccharides, and SCFAs offer promising avenues for preventing and treating food allergies. Human milk oligosaccharides (HMOs) and maternal dietary polyphenols transferred through breast milk exemplify a natural implementation of this synergistic strategy. HMOs such as 2′-fucosyllactose and lacto-*N*-fucopentaose promote butyrate production through fermentation of lacto-*N*-neotetraose and related structures, while dietary polyphenols simultaneously modulate maternal and infant gut microbiomes [129,130]. Intervention studies combining specific oligosaccharide–polyphenol pairings with OIT have shown higher desensitization rates, fewer adverse reactions, and improved long-term tolerance compared with OIT alone. Optimal combinations appear to pair short-chain FOSs or GOSs for rapid bifidogenic effects with phenolic acids or flavonoids that exhibit both epitope-masking properties (through covalent binding to allergenic proteins) and microbiome-modulatory activities [131,132,133]. However, several critical considerations temper these promising findings. Individual variability in microbiome composition, genetic factors influencing SCFA receptor expression, and differences in polyphenol-metabolizing capacity create substantial heterogeneity in treatment responses [114]. Only approximately 1% of healthy human gut metagenomes contain the complete genetic repertoire required for efficient flavonol catabolism to bioactive metabolites such as 4-Hydroxyphenylacetic acid, suggesting that not all individuals may equally benefit from polyphenol-based interventions without the concurrent provision of appropriate metabolizing bacteria [130,134]. Furthermore, dose–response relationships require careful optimization.

### 3.2. Probiotics Strategy

Probiotics play a critical role in managing food allergies by exerting anti-allergic effects through multiple complementary mechanisms, including direct immunomodulation, competitive exclusion of pathogens, production of antimicrobial compounds, and modulation of the endogenous microbiota composition and function [135]. At the cellular level, probiotic bacteria interact with intestinal epithelial cells and immune cells via pattern recognition receptors, particularly TLRs, which activate intracellular signaling cascades involving the nuclear factor-κB (NF-κB) and MAPK pathways that regulate cytokine secretion and immune cell differentiation [136].

Beyond direct interactions with immune cells, probiotics reshape the structure and function of the resident microbiota via productions of SCFAs [136]. SCFAs exert anti-allergic effects through dual signaling modalities: activation of GPCRs, and inhibition of histone deacetylases (HDACs), both of which profoundly influence immune cell differentiation, activation, and function [125,137,138]. SCFAs bind to and activate multiple GPCRs expressed on intestinal epithelial cells and immune cells, including GPR41, GPR43, GPR109A, GPR65, and Olfr78. GPR43 and GPR41 respond to SCFAs like acetate, propionate, and butyrate with varying affinities, whereas GPR109A binds selectively to butyrate and niacin but not to acetate or propionate. When SCFA binds, these receptors activate mitogen-activated protein kinase, extracellular signal-regulated kinase (ERK), and AMP-activated protein kinase (AMPK) signaling pathways [139,140]. In intestinal epithelial cells, SCFA-mediated activation of GPR43 and GPR41 induces expression of tight junction proteins (occludin, ZO-1), antimicrobial peptides, and chemokines that orchestrate immune cell recruitment and enhance epithelial barrier integrity [138]. This dual action prevents allergen translocation across the intestinal barrier and modulates local immune responses. GPR43 activation also suppresses pro-inflammatory cytokine secretion by inhibiting NF-κB signaling [139].

Early life colonization patterns by Bifidobacterium and Lactobacillus species constitute a second, clinically important functional cluster associated with allergy risk [141]. Several studies indicated that infants with a higher relative abundance of bifidobacteria are less likely to develop allergic sensitization, and that early colonization by lactobacilli is associated with more favorable allergy outcomes in later childhood [142]. Mechanistic work shows that selected Bifidobacterium strains can condition DCs toward a tolerogenic phenotype, increase IL-10 production, and drive the expansion of Foxp3+ Tregs, while also supporting IgA production at mucosal sites [143]. Lactobacillus species similarly modulate T-cell polarization, skewing responses away from Th2 dominance and toward balanced or regulatory profiles, with effects that are particularly pronounced when exposure occurs during the perinatal window of immune maturation. These immune effects complement the metabolic roles of butyrate producers by acting “upstream” at the level of antigen presentation and T-cell activation [142]. Host and dietary factors that foster these taxa provide a microbiota-level explanation for known protective exposures [141]. Exclusive breastfeeding and the supply of human milk oligosaccharides shape the initial microbiota toward Bifidobacterium-dominated communities, which in turn influence T-cell development and reduce the likelihood of early allergic sensitization.

#### 3.2.1. Generic Probiotics

Probiotic interventions using generic formulations are essential for achieving clinically meaningful changes in gut microbiota and immune regulation [144]. For instance, *Lactiplantibacillus plantarum* isolates have been shown to reduce IgE levels, suppress Th2 cytokines (IL-4, IL-5, and IL-13), enhance anti-inflammatory mediators (IL-10, IFN-γ, and TGF-β), and upregulate tight junction proteins, thereby linking symptom improvement to barrier restoration [145,146,147]. Other Lactobacillus strains, such as *Lactobacillus reuteri*, induce DCs to produce the anti-inflammatory IL-10 and promote the development of FoxP3-positive Tregs in the mesenteric lymph nodes and spleen; on the other hand, *Lactiplantibacillus plantarum* strains suppress Th2 cytokines and enhance IFN-γ production, shifting the immune balance from Th2 toward Th1 and regulatory phenotypes [148,149]. Interestingly, *Faecalibacterium prausnitzii* and *Roseburia intestinalis* probiotics enhance barrier function primarily through the production of SCFAs, like butyrate [44]. Butyrate selectively upregulates claudin-3 and claudin-4 protein expression at physiological concentrations (1–2 mM), and prevents lipopolysaccharide-induced downregulation of these proteins in intestinal epithelial cells [150]. Mechanistically, butyrate restores the connection between zonula occludens-1 and occludin proteins, thereby stabilizing tight junction assembly and increasing transepithelial electrical resistance. Butyrate also suppresses claudin-2, a pore-forming tight junction protein that increases paracellular permeability, through IL-10 receptor alpha-dependent and histone deacetylase inhibition pathways [151]. Moreover, butyrate conditions DCs to acquire tolerogenic phenotypes characterized by reduced expression of co-stimulatory molecules (CD80, CD86), decreased secretion of pro-inflammatory cytokines (IL-12, TNF-α), and increased production of anti-inflammatory mediators (IL-10, retinoic acid). These butyrate-conditioned DCs exhibit enhanced capacity to prime type 1 regulatory (Tr1) cells independently of TGF-β signaling, relying instead on increased expression of latency-associated peptide (LAP) and membrane-bound TGF-β [152].

Interestingly, Bifidobacterium species, particularly *Bifidobacterium bifidum*, upregulate occludin gene transcription and protein expression through sequential activation of the TLR-2/TLR-6 receptor complex, IRAK-1 phosphorylation, and apical membrane recruitment of the adapter protein TOLLIP, without affecting other tight junction components [153]. However, *Clostridium butyricum* protects intestinal barrier function via upregulating claudin-1, occludin, and ZO-1 while downregulating claudin-2 protein as observed in experimental colitis models [154]. These strain-based effects on tight junction proteins provide a mechanistic rationale for selecting probiotics to prevent food allergy. In a similar manner, the *Akkermansia muciniphila* probiotic strain enhances the intestinal barrier integrity through multiple mechanisms: *(a)* extracellular vesicles derived from *Akkermansia muciniphila* activate the AMPK signaling pathway in intestinal epithelial cells, which *(b)* induces the expression of occludin, claudin-5, and *(c)* zonula occludens proteins while reducing lipopolysaccharide-induced permeability [155]. Beyond direct effects on tight junctions, *Akkermansia muciniphila* maintains mucus layer turnover and stimulates goblet cell maturation through a TLR2-dependent pathway, ensuring a dynamic protective layer that prevents allergen contact with epithelial cells [156]. Another probiotic, *Faecalibacterium prausnitzii*, is a dominant butyrate producer in the healthy colon and has been shown to maintain the Th17-Treg balance and ameliorate experimental colitis by inhibiting histone deacetylase 1 and dampening c-Myc-driven metabolism in T cells [157]. Through this mechanism, butyrate derived from *Faecalibacterium prausnitzii* promotes Foxp3 expression, supports the differentiation and stability of Tregs, and limits pro-inflammatory Th17 response, thereby contributing to an immune environment that is more likely to tolerate food antigens than to mount IgE-mediated reactions [158]. However, butyrate derived from *Faecalibacterium prausnitzii* acts more directly on T cells, promoting Foxp3 expression and a Treg-dominant profile rather than Th17 or Th2 skewing the immune response [157,159]. Similarly, bifidobacteria and lactobacilli probiotics during early life shape mucosal DCs programming and T-cell conditioning toward tolerance, lowering the risk of IgE-mediated sensitization and helping consolidate long-term immune homeostasis [160].

#### 3.2.2. Specific Probiotics

Rather than generic probiotics, several specific strains have been shown to reduce allergic responses and promote immune tolerance to food allergens (Table 3). For example, *Lactiplantibacillus plantarum HM-22* shifted immune responses away from IL-4-driven inflammation by upregulating anti-inflammatory cytokines and gap junction proteins [148]. Similarly, other strains, such as *Bifidobacterium animalis KV9* and *Lactobacillus vaginalis FN3*, restore the Th1/Th2 balance and improve allergy outcomes, underscoring the importance of precise strain selection over generic supplementation [161,162,163].

**Table 3:** A list of specific probiotic strains and their role in enhancing gut microbiota.

Probiotic Strain	Targeted Food Allergy	Target Species	Outcome	Reference
*Lactobacillus casei Zhang* (LcZ)	Tropomyosin	Mouse	LcZ treatment reduced allergic symptoms, preserved gut integrity, and minimized tissue damage.	[164]
*Bifidobacterium longum subsp. longum 51A (BL51A)*	Ovalbumin	Mouse	BL51A-treated mice showed reduced weight loss and allergic symptoms.	[163]
*Propionibacterium freudenreichii CIRM-BIA129 (Pf129)*	Wheat gliadin	Mouse	Mice showed reduced anaphylaxis, lower allergen-specific IgE levels, serum mMCPT-1 levels, Th2 responses, and ILC2 activation, indicating decreased allergic inflammation.	[165]
*Lactobacillus plantarum A56*	Ovalbumin	Mouse	Oral *L. plantarum* A56 reduced allergic symptoms and lung inflammation, restored intestinal villi, and improved microbial diversity. It suppressed Ovalbumin-specific IgE and IgG1 while increasing IgG2a, thereby inhibiting Th2 responses and supporting gut health.	[147]
*Lactobacillus plantarum JC7*	Ovalbumin	Mouse	*L. plantarum* JC7 can prevent food allergies by correcting Th1/Th2 imbalances and modifying disordered intestinal microbiota.	[166]
*Akkermansia muciniphila BAA-835*	Ovalbumin	Mouse	*A. muciniphila* BAA-835-treated mice showed reduced weight loss and lower IgE and IgG1 levels, indicating diminished allergic response. Histology revealed less inflammation and tissue damage, with a marked decrease in eosinophil- and neutrophil-associated enzyme activity, key drivers of allergic inflammation.	[167]
*Lactobacillus rhamnosus GG*	Peanut	Human	In children, combining peanut protein with probiotics reduced skin-test reactions and peanut-specific IgE, while increasing IgG4, which is associated with tolerance. Side effects were primarily mild (e.g., stomach pain, mild allergic reactions), although a few were moderate or severe (including anaphylaxis), which were managed successfully. No cases of eosinophilic esophagitis were reported.	[168]
*Lactiplantibacillus plantarum HM-22*	α-lactalbumin (α-LA)	Mouse	Mice treated with this probiotic showed significant weight loss and increased levels of anti-inflammatory cytokines, which are associated with immune tolerance and suppression of allergic inflammation.	[148]
*Lactiplantibacillus plantarum YIT 0132 (LP0132)*	Cow milk allergy	Human	The probiotic showed a significant increase in β-lactoglobulin-specific IgG4, associated with tolerance development, and a notable reduction in IL-5 and IL-9, key cytokines that trigger allergic inflammation.	[149]
*Lactobacillus rhamnosus GGA*	Cow milk allergy	Human	Incorporating *L. rhamnosus* GGA into a cow milk-free diet resulted in a significant improvement in various gastrointestinal and behavioral symptoms in infants with cow milk allergy over four weeks.	[169]
*Lactobacillus rhamnosus* fermented milk (PFM)	Ovalbumin	Mouse	Feeding Lactobacillus rhamnosus fermented milk to mothers during the suckling period and to the offspring after weaning reduced clinical allergy in OVA-sensitized neonatal mice, lowered specific IgG/IgG1, and shifted immune responses from Th2 to Th1, with the strongest protection observed during the suckling period.	[170]

#### 3.2.3. Combination of Probiotics

Beyond individual strains, microbial consortia (associations of symbiotically interacting microorganisms from various species that interact through functional complementarity) and synbiotics (combinations of probiotics and prebiotics designed to work together) have shown promise in enriching the gut microbiota to combat food allergies (Table 4). For instance, combining a butyrate-producing commensal with its substrate restored gut function in dysbiosis, an imbalance in the microbial consortia living in the body [171]. A synbiotic of *Anaerostipes caccae* and lactulose increased colonic butyrate, reduced IgE and histamine, protected against anaphylaxis, and expanded Tregs in models mimicking infant allergy and post-antibiotic dysbiosis [106]. Probiotics can also enhance desensitization protocols, such as low-dose milk OIT combined with heat-killed *L. plantarum LP0132*, which increased β-lactoglobulin-specific IgG4, decreased IL-5/IL-9, and shifted the microbiota toward Lachnospiraceae, significantly improving milk tolerance by week 24 [149].

Selecting the appropriate strain is only part of the design; pairing it with a proper prebiotic makes the probiotic an additional important factor [172]. *Lactobacillus* with FOS, hydrolyzed casein with *Lactobacillus rhamnosus* GG (LGG), or traditional Dahi with *Lactobacillus/Bifidobacterium* have shown more pronounced and sustained benefits compared to either component alone [173]. The timing of probiotic administration is critical. Providing them during early life led to greater changes in immune response [174]. This aligns with a clinical meta-analysis that highlights benefits when probiotics are introduced during pre- and postnatal periods [175]. Probiotic formulations that produce tolerogenic signals near regulatory limits require precise dosing, strict containment, and thorough safety checks before they can be widely used [176].

**Table 4:** A list of combinations of probiotics/synbiotics in enhancing gut microbiota for the management of food allergies.

Probiotic/Synbiotic	Targeted Food Allergy	Target Species	Outcome	Reference
Probiotic mixture (P5: *Lactococcus lactis KF140*, *Pediococcus pentosaceus KF159*, *Lactobacillus pentosus KF340*, *Lactobacillus paracasei 698*, and *Bacillus amyloliquefaciens 26N*)	Ovalbumin	Mouse	P5 treatment significantly reduced Ovalbumin-specific IgE, suppressed Th2 and Th17 cytokines, and upregulated Th1 cytokines. This shift indicates restoration of the Th1/Th2 balance, a key factor in mitigating allergic responses.	[177]
Synbiotic *(Anaerostipes caccae and Lactulose)*	Cow milk allergy	Mouse	The synbiotic formulation restored colonic butyrate levels, protected against anaphylaxis, and promoted regulatory immune responses by suppressing inflammation and Th2 cytokines in mice.	[106]
Probiotic (*Bifidobacterium animalis KV9*, and *Lactobacillus vaginalis*)	Ovalbumin	Mouse	This combination of strains activated TLR4 via beneficial probiotics, thereby modulating immune responses. They upregulated MyD88 and IRF-1 in the spleen, thereby enhancing Th1 cells implicated in immune regulation and suppressing IRF-4, a transcription factor associated with allergic reactions.	[161,162]
Probiotic *(Lactobacillus rhamnosus ŁOCK 0900*, *Lactobacillus rhamnosus ŁOCK 0908*, and *Lactobacillus casei ŁOCK 0918*)	Cow milk allergy	Human	A 30% or more reduction in Atopic dermatitis (SCORAD scores) was observed primarily in children with allergy-related IgE antibodies.	[178]
Probiotic (*Bifidobacterium longum KACC 91,563* and *Enterococcus faecalis KACC 91532*)	Ovalbumin	Mouse	*B. longum* KACC 91563 was more effective in improving the food allergy symptoms than *Enterococcus faecalis* KACC 91532, which showed no effect. *B. longum* released extracellular vesicles that selectively eliminated mast cells responsible for allergic reactions without disrupting overall immune function.	[179]
Probiotic *(Bifidobacterium longum* subsp. *infantis*, *Lactobacillus acidophilus, Enterococcus faecalis,* and *Bacillus cereus)*	Ovalbumin	Mouse	The probiotic mixture helped cesarean-section-born rats early in life by reducing allergic responses by calming overactive Th2 responses that drive allergic symptoms.	[180]
Probiotic (*Lactobacillus acidophilus AD031*, *Bifidobacterium lactis AD011*)	Ovalbumin	Mouse	*B. lactis* AD011 and *L. acidophilus* AD031 have shown the potential to prevent or reduce allergic reactions.	[181]
Probiotic (*Lactobacillus rhamnosus GG* and *Bifidobacterium animalis* spp. *lactis BB-12*)	General food allergy (Non-specific)	Human	The combination alleviated mild food allergy symptoms in young children by reducing digestive issues. Blood test showed decreased IL-17, increased IL-10, and reduced IgE level, with effects persisting for months after treatment.	[182]
Probiotic (Dahi containing *Lactobacillus acidophilus LaVK2* and *Bifidobacterium bifidum BbVK3*)	Whey protein from cow’s milk	Mouse	Dietary probiotic Dahi reduced allergic reactions in whey protein-sensitized mice by shifting immunity from Th2 toward Th1 responses.	[183]

#### 3.2.4. Genetically Engineered Probiotics

Engineered probiotics, also referred to as live biotherapeutic products, are among the most promising innovations in modern microbiology. These microorganisms are intentionally designed to perform targeted therapeutic functions within the human body, such as detoxifying harmful compounds, delivering antigens, or modulating immune responses [184]. Unlike conventional probiotics that rely on naturally occurring strains, engineered probiotics incorporate customized genetic elements or synthetic pathways, making them more versatile but also introducing additional safety considerations [86]. Their ability to replicate, evolve, and interact with the native gut microbiota confers significant benefits but also poses risks distinct from those associated with traditional probiotics [185]. Even strains previously classified as generally recognized as safe (GRAS) have occasionally caused infections in vulnerable individuals [186], underscoring the need for rigorous safety assessments of genetically modified organisms [187]. In recent years, several engineered probiotics have been developed specifically to help manage food allergies (Table 5).

**Table 5:** A list of recombinant probiotics in enhancing gut microbiota for the management of food allergies.

Recombinant Probiotics	Type of Food Allergen Expressed	Target Species	Outcome	Reference
*Lactococcus lactis NZ3900/pNZ8149*-NapA and *L. lactis NZ3900/pNZ8149*	Ovalbumin	Mouse	Recombinant *Lactococcus lactis* supplementation reduced diarrhea in mice, lowered IgE, increased Ovalbumin-specific IgG, decreased IL-4, and boosted IFN-γ expression.	[188]
*Lactococcus lactis*	Ara h 2.02	Mouse	Oral administration of *Lactococcus lactis* expressing Ara h 2.02 can suppress the allergic immune responses in sensitized mice.	[189]
*Lactococcus lactis*	Ara h2	Human cells	The mimotopes elicited minimal allergic reactions and promoted a balanced immune response by increasing IFN-γ levels.	[190]
*Lactococcus lactis-rm IL10*	β-lactoglobulin	Mouse	Engineered *Lactococcus lactis* that secretes IL-10 reduced anaphylaxis and lowered IgE/IgG1 levels; increased gut IgA and IL-10 production in Peyer’s patches and plasma, which helped in preventing allergic reactions and sensitization.	[191]
*Lactococcus lactis*	β-lactoglobulin	Mouse	Recombinant *Lactococcus lactis* delivering BLG restored immune balance and prevented cow milk allergy sensitization by reducing allergen-specific IgE and enhancing IgG2a and IFN-γ.	[192]
*Lactococcus lactis MG1363*	Ara h2	Human-derived serum/antibodies	*Lactococcus lactis* efficiently produces full-length, active Ara h 2 with natural-like immune reactivity, enabling safer, standardized allergen immunotherapy.	[193]
*Lactococcus casei BL23*	β-lactoglobulin	Mouse	Recombinant probiotics induced IFN-γ (Th1) and mild IL-5 (Th2) responses.	[194]
*Lactococcus lactis MG1363*, *Lactococcus lactis NZ9000*	Ara h2	Mouse	Engineered *Lactococcus lactis* secreting Ara h 2 reduced IgE, enhanced IgG2a and IgA, and promoted Treg development.	[195]
*Lactococcus lactis MG1363*	Ovalbumin	Mouse	Oral delivery of antigens via recombinant *Lactococcus lactis* promotes antigen-specific tolerance by inducing adaptive Tregs, offering a promising strategy for treating allergic diseases.	[196]
Heat-killed *E. coli* engineered to produce mutated Ara h1, 2,3 (HKE-MP123)	Peanut hypoallergen	Mouse	Rectal delivery of HKE-MP123 induced durable desensitization in peanut-allergic mice, most pronounced at higher doses, by suppressing Th2 and enhancing Th1/Treg responses.	[197]
*Lactococcus lactis MG1363* *NZ9000*	β-lactoglobulin	Mouse	Achieved safe mucosal immune activation with *Lactococcus lactis* delivering BLG, eliciting strong mucosal IgA without systemic IgE, indicating reduced allergenicity.	[198]

#### 3.2.5. Synbiotics

The clinical translation of synbiotic approaches in food allergy management has shown promising outcomes, with probiotic-supplemented OIT demonstrating superior efficacy compared with allergen exposure alone [33]. In the landmark probiotic peanut OIT study, combining *Lactobacillus rhamnosus GG* with peanut OIT resulted in 82 percent sustained unresponsiveness, compared with 3.6 percent in the placebo group, and immune profiling revealed reduced peanut-specific IgE, elevated IgG4, and lasting tolerance at four-year follow-up [199]. Mechanistically, this benefit likely stems from the probiotic’s capacity to modulate both local gut immunity and systemic immune responses: *Lactobacillus rhamnosus GG* enhances tolerogenic DC function, promotes IL-10-producing Tregs, increases secretory IgA production, and shifts the microbiota toward SCFA-producing families [95]. Low-dose milk OIT combined with heat-killed *Lactiplantibacillus plantarum* showed similar mechanistic integration, with increased beta-lactoglobulin-specific IgG4, decreased IL-5 and IL-9, and a microbiota shift toward butyrate-producing taxa, significantly improving milk tolerance by week 24 [141]. These findings establish that food-derived probiotic and prebiotic interventions do not merely “add biomass” to the gut ecosystem but rather orchestrate coordinated changes in microbial composition, metabolism, and immune regulation that collectively promote tolerance to food allergens [157].

## 4. Clinical Trials

Translating microbiota-mediated immunomodulation from preclinical models to clinical practice has validated core mechanistic principles while highlighting a persistent gap between bioengineering potential and regulatory-approved therapies [135,200]. Clinical investigations to date have overwhelmingly used generic probiotic strains rather than engineered probiotics, reflecting regulatory caution, manufacturing complexity, and unresolved safety concerns about genetic stability and horizontal gene transfer [155,201]. This conservative trajectory offers an opportunity to assess whether naturally occurring probiotic interventions recapitulate the mechanistic effects, including tight junction reinforcement, SCFA production, Treg expansion, and DC tolerization, observed in preclinical studies using oligosaccharide additives, polyphenols, and engineered strains [151,202]. For instance, one of the probiotic-based peanut OIT (abbreviated as PPOIT) studies provides convincing clinical evidence that microbiota modulation enhances the acquisition of peanut tolerance in humans [203]. In this randomized controlled trial, which involved 62 children aged 1–10 years, the high-dose *Lactobacillus rhamnosus GG* (2 × 10^10^ CFU daily) combined with peanut OIT up to 18 months resulted in 82% sustained unresponsiveness after 2–5 weeks of peanut avoidance, compared with 3.6% in placebo recipients [204]. A four-year follow-up study demonstrated the durability of PPOIT, wherein 67% of probiotic-OIT participants regularly consumed peanuts, compared with 4% of placebo recipients, and there was a persistent rise in peanut-specific IgG4/IgE antibody ratios that indicated promising immune reprogramming toward peanut tolerance rather than transient desensitization [205]. Mechanistically, these clinical results align with key principles of allergen desensitization and tolerance: reducing systemic allergen-specific IgE, increasing Tregs that produce IL-10 and TGF-β, and enhancing allergen-specific IgG4 antibodies that block allergen binding to mast cells and compete with IgE [136,157,206]. However, the precise contribution of *Lactococcus rhamnosus GG* remains unclear, as a subsequent phase 2b trial (PPOIT-003) comparing probiotic-based peanut OIT (46% sustained unresponsiveness, meaning they do not react against peanut even after stopping OIT) with peanut OIT alone (51% sustained unresponsiveness) showed no significant additive benefit, suggesting that probiotic efficacy may be strain-specific, dose-dependent, or contingent on baseline microbiota composition [203,207,208]. This discrepancy highlights a significant translational hurdle: although preclinical studies show clear mechanisms by which particular bacterial strains influence tight junction proteins, SCFA levels, and immune cell types, applying these findings clinically demands identifying responder phenotypes. This involves baseline microbiota profiling, metabolomic signatures, and immune biomarkers to facilitate precise patient stratification [209,210].

Similarly, for cow’s milk allergy OIT, clinical investigations have directly linked specific bacterial taxa to the acquisition of sustained unresponsiveness [211]. In a multicenter Japanese study of 28 school-age children undergoing milk OIT for 12 months, followed by 2 weeks of milk avoidance, baseline and treatment fecal Bifidobacterium abundance was significantly associated with achieving sustained unresponsiveness. Children who achieved sustained unresponsiveness (5 of 6 tested, 83.3%) exhibited a higher relative abundance of Bifidobacterium species, particularly *Bifidobacterium pseudocatenulatum*, compared with non-responders [210]. Fecal metabolomic profiling revealed that sustained unresponsiveness was associated with distinct metabolite profiles, including elevated levels of specific amino acids and altered concentrations of SCFAs. The study identified gut microbiota composition and fecal metabolites as key clinical and environmental factors associated with the development of sustained unresponsiveness during milk OIT, providing mechanistic insight into the microbiota-immune axis during tolerance induction [149,212]. Similarly, in egg allergy OIT, the outcomes also exhibit microbiota-dependent patterns, although direct microbiota-profiling data remain limited. Probiotic-supplemented egg and milk OIT using *Lactobacillus rhamnosus GG* achieved high rates of sustained unresponsiveness, and mechanistic studies suggest that probiotic adjuvants enhance tolerogenic immune responses by modulating DC function and expanding Tregs [210].

## 5. Safety, Risks and Future Aspects for Bioengineered Interventions for Food Allergy Management

The current landscape of microbiota-targeted interventions for food allergy management has demonstrated proof-of-concept efficacy across multiple modalities, including generic probiotics, oligosaccharide prebiotics, polyphenol conjugates, and recombinant probiotics, yet clinical translation remains constrained by a critical disconnect between preclinical mechanistic sophistication and therapies approved by regulators [199,213]. Plasmid-based expression systems commonly used in preclinical studies are segregationally unstable, with loss rates of 1–10% per generation in the absence of antibiotic selection [214]. In the human gut, where bacterial doubling times approximate 2–4 generations per day and antibiotic selection is impermissible, maintaining plasmid-borne allergen expression during a 14–90-day OIT induction phase requires stable chromosomal integration, auxotrophic dependencies, or selection-free plasmid maintenance systems [215,216]. A technological hurdle is the chromosomal integration via site-specific recombinases (attTn7, phage integrases), which provide single-copy, stable expression but complicate strain construction, reduce genetic flexibility for iterative optimization, and introduce integration-site-dependent transcriptional variability that affects allergen expression levels [217,218,219,220]. Alternative strategies employing partitioning systems (ParAB) and post-segregational killing modules (toxin–antitoxin cassettes) maintain episomal plasmids without antibiotic selection but face evolutionary pressure favoring escape mutants that inactivate toxin genes [221,222].

Recent advances in CRISPR-based genome editing enable scarless chromosomal integration with defined copy-number control; however, current protocols require multiple cloning steps and counter-selection markers (e.g., upp, thyA) that limit strain-engineering throughput [223,224]. Horizontal gene transfer to commensal or pathogenic gut bacteria represents the second biocontainment failure mode [225]. Conjugative plasmids can transfer at frequencies exceeding 10^−3^ transconjugants per donor cell under gut-like conditions, risking dissemination of antibiotic resistance markers, or allergen-encoding genes to the resident microbiota [226,227]. Another technical hurdle is that standard mitigation strategies, such as eliminating origin of transfer sequences, removing mobilization genes, and incorporating incompatibility determinants, reduce but do not eliminate horizontal gene transfer, as chromosomal DNA can undergo recombination-mediated transfer during bacterial conjugation or genetic transduction by temperate phages [225,228,229,230,231]. Engineering probiotic strains with defective recombination machinery prevents horizontal gene integration but compromises bacterial fitness and DNA repair capacity, creating selective pressure for reversion [232]. Recent innovations employ CRISPR-based conjugation blockers that target and cleave transferred DNA containing specific signature sequences, reducing transconjugant formation by 100-fold, but they require practical optimization for each probiotic chassis and add genetic complexity that compounds regulatory approval challenges [233].

Kill switches designed to eliminate engineered strains upon environmental escape or upon completion of treatment are evolutionarily unstable because strong negative selection against toxin expression acts against them [234]. Single-input, chemical-responsive kill switches based on toxin–antitoxin systems or nuclease expression accumulate escape mutants within 10–20 generations, as point mutations in sensor promoters, toxin-encoding sequences, or upstream regulatory elements confer an immediate survival advantage [235,236,237,238]. Even with optimized kill switches, regulatory agencies will likely mandate layered biocontainment combining genetic safeguards with auxotrophic dependencies (thymidine, diaminopimelic acid, or mucin-derived sugars unavailable outside the gut) and temperature-sensitive replication origins that prevent growth at environmental temperatures [239,240,241].

Regulatory frameworks for live biotherapeutic products constitute the third major translational barrier [242]. FDA guidance documents specify requirements, including complete genome sequencing to verify the absence of virulence factors and mobile genetic elements; genetic stability testing under good manufacturing practice fermentation conditions for 50+ generations; demonstration that engineered traits do not alter colonization dynamics or microbiome composition; assessment of reversion frequency for auxotrophic dependencies; and post-market pharmacovigilance monitoring for persistence beyond the intended treatment duration and for horizontal gene transfer in clinical populations [243,244,245]. Thus, the chromosomal integration of genetic constructs is generally preferred over plasmid-based expression to reduce mobility and gene transfer risks [246]. Developers are also encouraged to avoid antibiotic selection markers, incorporate nutrient dependencies (auxotrophies), and design multilayered containment strategies, such as redundant kill switches or “gene-erase” systems [247,248]. Another technical hurdle is that extending this safety profile to food allergy applications requires allergen-specific immunological monitoring to distinguish therapeutic desensitization from inadvertent sensitization, particularly during dose escalation, when engineered bacteria transiently colonize and express allergens at mucosal surfaces [249,250]. The regulatory requirement for comparator clinical trials demonstrating superiority over standard OIT creates an economic disincentive [251,252,253,254]. Precision microbiota engineering is an emerging frontier that extends beyond single-strain probiotic interventions to encompass defined microbial consortia, targeted metabolite supplementation, and ecological niche engineering [255,256].

Multi-omics integration promises to decode responder phenotypes and enable predictive algorithms for microbiota interventions [257,258]. Another research direction comprises machine learning models integrating baseline microbiota composition (16S rRNA sequencing), metabolomics (untargeted Liquid Chromatography–Tandem Mass Spectrometry (LC-MS/MS)), immune phenotypes (flow cytometry for Tregs, DCs, and mast cell markers), genetic polymorphisms (HLA alleles, STAT6, and IL4R variants), and epigenetic modifications (FOXP3 TSDR methylation, IL-10 promoter histone acetylation) [259,260,261,262,263]. Further investigations are required for the comparative efficacy of defined bacterial consortia versus single-strain probiotics.

## 6. Conclusions

Current bioengineering efforts highlight the significant potential in manipulating the gut microbiome to prevent or treat food allergies. Achieving this potential will involve overcoming crucial translational challenges: maintaining the genetic stability of engineered probiotic strains, deploying reliable and evolution-resistant biocontainment systems, and creating regulatory policies that promote innovation while ensuring safety. Future advancements will rely on integrated approaches that merge mechanistic microbiome engineering with clinical immunology, ultimately paving the way for precise, scalable, and safe microbiota-centered treatments for allergies.

## Data Availability

No new data were created or analyzed in this study.

## Abbreviations

AAAAI	American Academy of Allergy, Asthma & Immunology
ABC	ATP-binding cassette
ACAAI	American College of Allergy, Asthma, & Immunology
ACT	Apple condensed tannin
ACLY	ATP citrate lyase
AIT	Allergen-specific immunotherapy
AMPK	AMP-activated protein kinase
APC	Antigen-presenting cell
BMDC	Bisdemethoxycurcumin
BTK	Bruton’s tyrosine kinase
CA	Chlorogenic acid
CAZyme	Carbohydrate-associated enzyme
CMA	Cow’s milk allergy
CNS2	Conserved non-coding sequence 2
COS	Chitosan oligosaccharide
CREB	cAMP response element-binding protein
CRISPR	Clustered Regularly Interspaced Short Palindromic Repeats
DCs	Dendritic cells
DP	Degree of polymerization
EGCG	Epigallocatechin gallate
EHCF	Extensively hydrolyzed casein formula
ERK	Extracellular signal-regulated kinase
FDA	Food and Drug Administration
FOS	Fructo-oligosaccharide
FOXP3	Forkhead box protein 3
GOS	Galacto-oligosaccharide
GPCR	G protein-coupled receptor
HAT	Histone acetyltransferase
HDAC	Histone deacetylase
HIF1α	Hypoxia-inducible factor 1Î±
HLA	Human leukocyte antigen
HMO	Human milk oligosaccharide
4-HPAA	4-hydroxyphenylacetic acid
IgE	Immunoglobulin E
IgG	Immunoglobulin G
IL	Interleukin
IFN-γ	Interferon gamma
IP-10	Interferon gamma-inducible protein-10
IRAK-1	Interleukin-1 receptor-associated kinase 1
LAP	Latency-associated peptide
LAT	Linker for activation of T cells
MAPK	Mitogen-activated protein kinase
mMCP-1	Mouse mast cell protease-1
mTOR	Mechanistic target of rapamycin
NF-κB	Nuclear factor kappa B
NLRP3	NOD-like receptor family pyrin domain-containing 3
OIT	Oral immunotherapy
PAZyme	Polyphenol-associated enzyme
PBMC	Peripheral blood mononuclear cell
PGE2	Prostaglandin E2
PIK3D	Phosphatidylinositol 3-kinase delta
PPOIT	Probiotic and Peanut Oral Immunotherapy
RETPS	Red-edge tea polysaccharides
RMD	Raffinose/melibiose oligosaccharides
RPTOR	Regulatory-associated protein of mTOR
SCFA	Short-chain fatty acid
SlpB	Surface layer protein B
SLIT	Sublingual immunotherapy
STAT6	Signal transducer and activator of transcription 6
SYK	Spleen tyrosine kinase
Tfh	T follicular helper
TGF-β	Transforming growth factor beta
TIM4	T-cell immunoglobulin and mucin domain containing 4
TNF-α	Tumor necrosis factor alpha
TOLLIP	Toll-interacting protein
Tregs	Regulatory T cells
TSA	Trichostatin A
TSDR	Treg-specific demethylated region
ZO-1	Zonula occludens-1
